# Permanent Paddle-lead Trial for Spinal Cord Stimulation

**DOI:** 10.7759/cureus.2645

**Published:** 2018-05-18

**Authors:** Jonathan J Lee, Richard K Simpson, Brian Dalm

**Affiliations:** 1 Department of Neurosurgery, Houston Methodist Neurological Institute, Houston, USA

**Keywords:** spinal cord stimulation, trial, chronic pain, failed back surgery syndrome, paddle electrode, paddle-lead trial

## Abstract

Objectives

A significant number of chronic pain patients rely on spinal cord stimulation (SCS) for treatment of their intractable pain. A screening trial using percutaneous electrodes is an integral step for predicting a successful treatment course with a permanent SCS system. Most of these trials are performed in an outpatient ambulatory surgical center and some in the office setting. However, there are select patients who are considered poor candidates for percutaneous trials. We present the initial report of patients who have received surgical implantation of permanent paddle-leads for SCS trials at our institution and show that this was a safe and effective alternative for our patients who could not undergo percutaneous trials.

Methods

We retrospectively reviewed the hospital charts of 12 patients who underwent permanent surgically-implanted paddle-lead trials from 2014 to 2017. Success was considered positive with a 50% reduction in pain rating. If positive, patients were brought back to the operating room to have the implanted leads connected to an internalized pulse generator.

Results

All 12 patients met the criteria for a successful trial. Only one patient had his SCS system surgically removed after nine months. None of our patients reported or returned with paddle-lead migrations or infections.

Conclusions

We report that surgically-implanted paddle-lead trials were a safe and effective alternative to percutaneous trials in our patients who were deemed poor candidates for percutaneous trials. No complications occurred and all of our patients received a second surgery for internalization of the SCS system. Patients who have previously failed percutaneous trials may be false-negatives to SCS.

## Introduction

A significant number of patients with chronic pain, including complex regional pain syndrome (CRPS) and failed back surgery syndrome (FBSS), rely on spinal cord stimulation (SCS) for the treatment of their intractable pain. SCS is a well-established treatment for a variety of neuropathic pain syndromes [1-6].

A screening trial of SCS using percutaneous electrodes is an integral step for predicting a successful treatment course with a permanent spinal cord stimulator. Most percutaneous trials are performed in an outpatient ambulatory surgical center and some in the office setting. However, there are select patients who are considered poor candidates for these percutaneous trials. For instance, patients with a history of multiple spine surgeries, specifically in the upper lumbar or lower-to-mid-thoracic spine, can develop epidural scar tissue that makes the passing of trial leads technically difficult or impossible. Percutaneous trials for patients with instrumentation from prior thoracic fusions are also often unsuccessful due to the hindrance of the instrumentation to the passage of the leads. Additionally, patients with high-grade scoliosis can make percutaneous trials unsuccessful due to the negative effect of the severe curvature of the spine on the course of the leads.  

An alternative for patients who are deemed poor candidates for percutaneous trials is screening using surgical implantation of permanent paddle-leads. We present a report of 12 patients at our institution who underwent permanent surgically-implanted paddle-lead trials for SCS.  

## Materials and methods

We retrospectively reviewed the hospital charts of 12 patients who underwent permanent surgically-implanted paddle-lead trials from 2014 to 2017. All patients were evaluated by pain management physicians who concluded that SCS was an appropriate treatment strategy. Records were reviewed to establish patient demographics.

All patients were referred to our outpatient neurosurgery clinic after they were deemed poor candidates for percutaneous trials. These patients were interviewed and examined in our neurosurgery clinic. Patients were regarded as good candidates for surgical implantation of SCS paddle-leads based on a history of chronic pain and report from their pain management physicians that a percutaneous trial had either failed or was assessed as technically too difficult. All patients agreed to SCS trials using permanent paddle-lead surgical implantation, and all patients provided consent for the procedure. Patients were taken to the operating room and were placed under general anesthesia.

After local anesthetic and a skin incision at the appropriate level, dissection was carried down to the spinous process. A rongeur was then used to trim the spinous process. The ligamentum flavum was opened and bilateral laminotomies were made with a combination of rongeurs. Leads were placed according to the pain distribution at either the cervical or thoracic level. For low back and leg pain, T8-9 with coverage extending across the T9-10 disc space was used with Medtronic leads (Medtronic, Inc., Minneapolis, MN). Boston Scientific (Boston Scientific, Inc., Valencia, CA) lead placement was generally utilized for T7-8. All leads were placed using C-arm fluoroscopy and placed in the dorsal epidural space along the anatomic midline. Once confirmed in the appropriate location, the lead tails were secured with silk sutures onto the spinous process below. The lead tails were then passed to an incision created in the upper lumbar region and connected to lead extensions, which were then externalized and connected to a pulse generator. 

Patients were evaluated over the next few days to determine the success of the trial. The exact number of days for each trial is seen in Table 1. As in traditional percutaneous SCS trials, a successful trial is deemed positive if the patients report that they have experienced greater than 50% pain relief from their baseline pain. Patients were evaluated after the trial by their referring physicians or pain management physicians. If positive, patients were brought back to the operating room to have the implanted leads connected to an internalized pulse generator.

**Table 1:** Patient Demographics and Results CPS: chronic pain syndrome; PLS: post-laminectomy syndrome; FBS: failed back syndrome; FNS: failed neck syndrome

Age (yrs) & Sex	Trial Year	Etiology	Distribution	Indication	Paddle Electrode	Location	Trial Length (days)	Trial Pass/Fail	F/U (mos)	Revision/ Removal
66 F	2014	CPS, PLS, FBS	Back & leg	Extensive spine surgery	Boston Scientific	Thoracic	5	Pass	1	N
69 M	2014	CPS, PLS, FBS	Back, hip, & leg	Extensive spine surgery; aborted percutaneous trial due to scarring	Boston Scientific	Thoracic	7	Pass	1	N
77 M	2014	CPS, PLS, FBS	Back & leg	Aborted percutaneous trial due to misplacement	Boston Scientific	Thoracic	9	Pass	1	N
50 M	2015	CPS, PLS, FBS	Leg & foot	Extensive spine surgery, scoliosis, hardware	Medtronic	Thoracic	5	Pass	1	N
60 M	2015	CPS, PLS, FBS	Back, hip, & leg	Extensive spine surgery, hardware	Boston Scientific	Thoracic	8	Pass	1	N
75 M	2016	CPS, PLS, FBS	Back & leg	Extensive spine surgery, hardware, scoliosis	Boston Scientific	Thoracic	5	Pass	X	N
34 M	2016	CPS, PLS, FNS, SCI	Arm & shoulder	Extensive spine surgery, hardware	Medtronic	Cervical	6	Pass	9	Y
60 F	2016	CPS, PLS, FBS	Neck, arm, back, & leg	Extensive spine surgery, scoliosis, hardware	Boston Scientific	Thoracic	8	Pass	1	N
30 F	2016	CPS, PLS, FBS	Back & leg	Extensive spine surgery, hardware	Medtronic	Thoracic	7	Pass	X	N
47 F	2016	CPS, PLS, FBS	Leg	Extensive spine surgery, scoliosis, hardware	Boston Scientific	Thoracic	11	Pass	1	N
53 M	2017	CPS, FBS, SCI	Thorax & waist	Extensive spine surgery, hardware	Medtronic	Cervical	7	Pass	1	N
43 F	2017	CPS, FBS, SCI	Hip, leg, foot	Extensive spine surgery, hardware	Medtronic	Thoracic	7	Pass	1	N

Postoperative pain for each patient was similar and comparable to standard surgical paddle-lead placement. Most often, postoperative pain from this procedure was controlled with Tylenol® #3 (Janssen-Ortho, Inc., Toronto, Ontario, Canada) (p.o. codeine/acetaminophen). 

## Results

Table 1 summarizes the patient demographics of the 12 patients who underwent permanent surgically-implanted paddle-lead trials for SCS at our institution from 2014 to 2017. The cohort consisted of five (41.7%) females and seven males (58.3%). The average age was 55.3 years; the youngest patient was 30 years old and the oldest patient was 77 years old. Most patients had multiple diagnoses per International Statistical Classification of Diseases and Related Health Problems (ICD)-10 coding and suffered from pain in multiple areas, including back, leg, hip, foot, neck, and arm. Specific diagnoses and distribution of the patients’ pain can be seen in Table 1.

Eleven patients could not undergo a percutaneous trial due to previous extensive spine surgery, and one patient had an aborted percutaneous trial due to misplacement. Of those who had extensive spine surgeries, nine had associated hardware in the spine, four had associated high-grade scoliosis, and one had an attempted percutaneous trial that was aborted due to scarring. 

Boston Scientific surgical paddle-leads were used in seven patients, and Medtronic surgical paddle-leads were used in five patients. Paddle-leads were placed in the thoracic region in 10 patients and in the cervical region in two patients.

All patients met the criteria for a successful trial – all patients underwent a second operation to have the paddle-leads connected to an internalized pulse generator.

For pain management and evaluation after the second operation (i.e., full implantation of the SCS system), all patients followed up with their pain management physicians. Ten out of 12 patients followed up in our neurosurgical outpatient clinic after this second operation to have their pain assessed and their incisional sites evaluated. All 10 patients reported a significant reduction in their pain compared to pre-operation and all incisions were without complications. Only one patient had his permanent SCS system surgically removed nine months after implantation due to complaints of a loss of therapeutic effect. None of our patients have reported or returned with evidence of paddle-lead migrations or infections.

## Discussion

Given the evidence of the effectiveness of spinal cord stimulation (SCS), all patients in whom SCS is indicated for their pain should have the opportunity of implantation. Implantation of an SCS system typically begins with a trial with percutaneous electrodes. However, some patients are not deemed adequate candidates for these percutaneous trials. In our cohort, 11/12 patients had previous back surgeries (i.e., laminectomies or fusions) that would have made percutaneous trials technically difficult.

Surgically-implanted paddle-leads are becoming a viable alternative for patients who are deemed poor candidates for percutaneous SCS trials. In 2014, Pahapill et al. published a report of 22 patients who received paddle-lead screening trials and concluded that the results were similar to those seen with standard percutaneous screening trials [7]. Pahapill et al. reported a success rate of 73%. All of our patients reported their pain to be decreased enough to receive a second surgery to have their systems fully-internalized. As our patient population expands, we do expect to find non-responders. We also acknowledge that our patients were previously screened by pain physicians to be excellent candidates for SCS.

Our patient sample size is currently too small to make definitive comparisons about the success of paddle-lead SCS over percutaneous lead trials. In a national retrospective review over a nine-year period, Huang et al. analyzed over 21,672 patients who underwent percutaneous trials for SCS [8]. Perhaps as we increase our volume of patients who are appropriate candidates for surgically-implanted trials, we can begin to make comparisons of the rates of success between our patients who undergo percutaneous and surgically-implanted trials.

Another limitation of our study is the short follow-up period that inhibits us from making substantial claims about the long-term success of permanent SCS after paddle-lead trials. Patients return to our clinic for their postoperative evaluation and then return for further evaluation if they are no longer experiencing the desired clinical benefits from the stimulation; however, it is possible that patients simply did not return to our clinic despite a lack of long-term success after permanent implantation.

We are in the process of implementing a system to follow-up on our paddle-lead trial patients in the long-term. We expect to find some patients who have permanent SCS systems that have lost therapeutic effect as our number of permanent lead trials expand and as our follow-up time lengthens, given that most failures of SCS occur early [9]. This may be due to differences in the location, lead shape, lead design, and pattern of lead placement inherent to paddle-leads (versus percutaneous leads), such as their conical lead shape and more variable epidural location.

Patients were given a week-long course of Tylenol #3 postoperatively. We acknowledge that postoperative pain medication can sometimes mask the effects of stimulation and that acute postoperative pain itself could have had a possible gating effect on the patients’ chronic pain [10]. However, we do not believe the addition of this analgesic in our patients who had undergone previous medication regimens significantly contributed to the overall success rate of the trials, and we believe acute postoperative pain eventually subsided to the point where they could accurately assess their chronic pain.

Despite the effectiveness of permanent surgically-implanted paddle-leads [11-12], this procedure is not without risks. The fact that the trial requires implantation of a plate electrode through laminotomies makes it a more invasive alternative and can be more distressing to some patients. Additionally, laminotomies have their own risks of infection, epidural hematomas [13], intraoperative root or spinal cord injury [14], or potential spinal instability, especially if the laminotomies need to be converted to laminectomies that extend past the intended single level. Moreover, there is an inherent risk that a surgically-implanted SCS paddle trial may not be successful, in that a patient may not receive a permanent SCS system.

Given the high success of our patients with paddle-lead trials, we wish to highlight the possibility that patients who have had failed percutaneous trials may be false-negatives to SCS. If a much larger study is done that shows a higher success rate of paddle-lead trials compared to success rates of percutaneous trials, the implication could be that patients who could actually benefit from SCS are being wrongly rejected as candidates after failed percutaneous trials. We hope to investigate this possibility in future studies.

## Conclusions

In this preliminary report, we report surgically-implanted paddle-lead trials were a safe and effective alternative to percutaneous lead trials in our patients who were deemed poor candidates for percutaneous trials. Although our sample size is too small to make conclusions about paddle-lead SCS success as a whole, no complications occurred during our trials and all of our patients received a second surgery for full internalization of the SCS system. More trials will need to be performed and a more robust protocol for follow-up will need to be instilled in order to provide more evidence for its effectiveness. Patients who have failed percutaneous trials may be false-negatives to SCS.

## References

[1] Geurts JW, Smits H, Kemler MA (2013). Spinal cord stimulation for complex regional pain syndrome type I: a prospective cohort study with long-term follow-up. Neuromodulation.

[2] Kumar K, Taylor RS, Jacques L (2007). Spinal cord stimulation versus conventional medical management for neuropathic pain: a multicentre randomised controlled trial in patients with failed back surgery syndrome. Pain.

[3] Kumar K, Taylor RS, Jacques L (2008). The effects of spinal cord stimulation in neuropathic pain are sustained: 24-month follow-up of the prospective randominzed controlled multicenter trial of the effectiveness of spinal cord stimulation. Neurosurgery.

[4] North RB, Kidd DH, Farrokhi F, Piantadosi SA (2005). Spinal cord stimulation versus repeated lumbosacral spine surgery for chronic pain: a randomized, controlled trial. Neurosurgery.

[5] North RB, Kumar K, Wallace MS (2011). Spinal cord stimulation versus re-operation in patients with failed back surgery syndrome: an international multicenter randomized controlled trial (EVIDENCE study). Neuromodulation.

[6] Cameron T (2004). Safety and efficacy of spinal cord stimulation for the treatment of chronic pain: a 20-year literature review. J Neurosurg.

[7] Pahapill PA (2014). Surgical paddle-lead placement for screening trials of spinal cord stimulation. Neuromodulation.

[8] Huang KT, Martin J, Marky A (2015). A national survey of spinal cord stimulation trial-to-permanent conversion rates. Neuromodulation.

[9] Hayek SM, Veizi E, Hanes M (2015). Treatment‐limiting complications of percutaneous spinal cord stimulator implants: A review of eight years of experience from an academic center database. Neuromodulation.

[10] Melzack R, Wall PD (1965). Pain mechanisms: a new theory. Science.

[11] North R, Kidd D, Davis C (1999). Spinal cord stimulation electrode design: A prospective randomized, controlled trial comparing percutaneous and laminectomy electrodes. Stereotact Funct Neurosurg.

[12] North RB, Kidd DH, Petrucci L, Dorsi MJ Spinal cord stimulation electrode design: a prospective, randomized, controlled trial comparing percutaneous with laminectomy electrodes: part II-clinical outcomes. Neurosurgery.

[13] Moufarrij NA (2016). Epidural hematomas after the implantation of thoracic paddle spinal cord stimulators. J Neurosurg.

[14] Jeon YH (2012). Spinal cord stimulation in pain management: a review. Korean J Pain.

